# Methylphenidate (Ritalin) does not improve exam performance in an experimental setting

**DOI:** 10.1007/s00213-025-06864-1

**Published:** 2025-08-05

**Authors:** Anke Sambeth, Monika Toth, Arjan Blokland

**Affiliations:** 1 Department Teaching and Innovation of Learning, Faculty of Psychology and Neuroscience, Maastricht University, PO Box 616, Maastricht, 6200 MD The Netherlands; 2 Department of Neuropsychology & Psychopharmacology, Faculty of Psychology and Neuroscience, Maastricht University, Maastricht, The Netherlands

**Keywords:** Methylphenidate, Exam performance, Memory, Student performance, Ritalin

## Abstract

**Rationale:**

Surveys indicate that about 10–20% of students use medicinal drugs to improve their exam performance. Whether these drugs really improve exam performance has not been examined in an experimental setting yet. This study tested the effects of methylphenidate (MPH; 20 mg) on exam performance either by giving the drug before studying for an exam (day 1, acquiring new information) or before the exam was taken (day 2, retrieving the information).

**Method:**

For this study, a double-blind placebo controlled between-subjects design was applied. The participants were randomly assigned to three groups that were given treatment on the two days: Placebo-Placebo (*n* = 25), MPH (*n* = 24), Placebo-MPH (*n* = 26). The exam contained multiple-choice questions (factual knowledge and inference questions) and open questions (inference).

**Results:**

The data showed that MPH did not improve the exam performance on the three types of questions. In addition, the average grade did not differ between the three groups and the number of participants failing or passing the exam did not differ.

**Conclusion:**

This is a first experimental study showing that MPH does not improve exam performance and should discourage students to take MPH during exam periods.

## Introduction

Several studies have shown that 10–20% of the student population use cognition enhancing drugs (CEDs) in order to pass their exams (e.g. Lengvenyte et al. 2016; McDermott et al. 2021; Ponnet et al. 2021; Sharif et al. 2021; Teter et al. 2005). There is also the belief amongst the student population that CEDs can enhance the academic performance (e.g., Vargo and Petroczi 2016). The percentage of using stimulants, such as methylphenidate (MPH), appears to be lower and estimated in the range of 4–8% of student populations (Cándido et al. 2020; Liakoni et al. 2015; Maier et al. 2013; Sussman et al. 2006; Teter et al. 2006; Van Zyl et al. 2017). Modafinil, which is used by a lower percentage of students has wake promoting properties and there are reports that modafinil enhances cognitive performance (Battleday and Brem 2015). For MPH, this effect is probably based on the attention enhancement that has been reported for ADHD patients (e.g., McKenzie et al. 2022), and reports showing that MPH can enhance cognitive performance in healthy young subjects (see Linssen et al. 2014).

However, the use of MPH is not without any dangers and reported side effects are related to cardiovascular issues, abuse potential, and even psychosis, depression and suicidal ideation (e.g., Lakhan and Kirchgessner 2012; Ragan et al. 2013; Ryst and Childress 2023). Apart from safety issues, there have been discussion related to the ethical aspects of the use of CEDs for academic performance (e.g., Maslen et al. 2014; Sahakian and Morein-Zamir 2011). Although these ethical concerns have been raised, it has not been investigated whether CEDs can actually improve academic performance.

Although various studies have shown positive effects of drugs on cognitive performance, the number of drugs that can improve cognitive performance in experimental studies is rather limited. More importantly, the drugs that have shown to improve cognition do not improve all cognitive functions. For example, MPH most consistently improves working memory and processing speed, and some studies reported improved long-term episodic memory (see Linssen et al. 2014). Fewer studies reported enhanced performance in verbal word learning and attention/vigilance tasks. These effects of MPH are assumed to related to its mechanism of action, inhibition of the catecholamines dopamine and noradrenaline reuptake which enhances catecholamine availability (Hannestad et al. 2010; Volkow et al. 1998). Another CED is modafinil, which is also used by students, but less frequently. In contrast to MPH, modafinil appears to improve performance in executive function and attention tasks and to a lesser consistent improvement in and learning and short-term memory (Battleday and Brem 2015). However, this was dependent on the complexity of the tasks used for these cognitive functions, with a lack of improvement in low complexity tasks. It should be noted that, although MPH and modafinil may improve cognitive performance in some domains as mentioned above, the cognition enhancing properties are limited to certain domains (Roberts et al. 2020). In addition, it has also been suggested that some of the effects of MPH could be attributed to enhanced energy and motivation (Ilieva et al. 2015). While coffee and tea also have cognition enhancing properties (attention and processing speed, see Anas Sohail et al. 2021; Fiani et al. 2021), and are freely accessible, these are not generally considered as CED.

Although experimental studies have shown that several drugs and natural products can enhance specific aspects of cognitive performance, no studies have examined the effects of these drugs on exam performance in an experimental setting yet. Therefore, we designed a study in which we tested the effects of MPH on exam performance. The study consisted of a learning phase, during which students had to learn texts on a non-familiar topic, and an exam phase. Since it is unknown whether MPH enhances the learning (acquisition of new information) or performance during an exam (retrieval of stored information), we gave MPH before learning or before taking the exam. In addition, the exam tested factual knowledge of the texts and contained questions that required inference of the learned texts. Based on the beneficial effects of MPH on memory, it was expected that MPH would also improve exam performance, either by improving encoding or retrieval of new information. Since the effects of MPH on cognition have been shown to be dependent on baseline dopamine levels, previous studies included a working memory task to control for individual differences in baseline levels (Mattay et al. 2000; Mehta et al. 2000; van der Schaaf et al. 2013). In this study, a digit-span task was included in order to control for these baseline differences.

## Method

### Participants

Seventy-five healthy young undergraduate university students (20 male, 55 female) with a mean age of 23.09 years were recruited by means of local advertisements. Prescreening occurred using a medical questionnaire on physical and mental health. The main inclusion criteria were age (18–35 years) and body mass index (18.5–30 kg/m2). Exclusion criteria were history or presence of mental or physical disorders, history of alcohol or drug use, ADHD, dyslexia, pregnancy, use of medication other than contraceptives, use of recreational drugs from 2 weeks before until the end of the experiment and consumption of more than 20 alcohol containing beverages per week. Participants were requested to abstain from any drug use 1 week prior to the test sessions and to not drink any alcoholic beverages 24 h prior to testing. All subjects gave written informed consent and received a financial reward for their participation. The study was approved by the local Medical Ethical Committee and in accordance with the Declaration of Helsinki.

### Design

The study was conducted according to a randomized, double-blind, placebo-controlled design. Participants were randomly assigned to one of the three treatment sequences: placebo on both days (PLA-PLA; *n* = 25), 20 mg MPH on day 1 and placebo on day 2 (MPH-PLA; *n* = 24), or placebo on day 1 and 20 mg MPH on day 2 (PLA-MPH; *n* = 26). The two test days were separated by 24 h.

### Treatment

In this study, a dose of 20 mg was chosen that was considered the average dose that is typically prescribed for ADHD patients. Studies also indicate that the usual MPH dose used by students is 20 mg (Finger et al. 2013/and internet such as reddit.com). Therefore, a 20 mg dose was chosen for the current study. The pills containing MPH and placebo were identical with regard to shape and color. The experimenters were blind to the treatment condition. Based on the maximum plasma concentration of MPH being reached one to three hours after oral ingestion (Leonard et al. 2004), participants were tested 80 min after administration of MPH or placebo on both test days.

### Heart rate and blood pressure

Since MPH can increase heart rate and blood pressure (e.g., Kimko et al. 1999), blood pressure (systolic and diastolic) and heart rate were measured before drug administration and 3 h later (after the end of the test).

### Exam

On test day 1, participants received four short texts (see Thiede et al. 2003) to study within 35 min. These texts discussed topics of naval warfare in WW2, Norse settlements in the Viking Age, cross-cultural communication, and the influence of alcohol on sleep. Then, 24 h later, on test day 2, they were asked to take an exam based on the texts. The exam consisted of a total of 60 questions, of which 48 were multiple-choice questions (12 per text) and 12 open-ended questions (3 per text). For each text, the multiple-choice questions were further subdivided into factual knowledge (6 questions) and inference questions (6 questions). The multiple choice questions were copied from an earlier study using these texts (Thiede et al. 2003). The 3 open-ended questions, also assessing inference and comprehension, were developed and first piloted by us before this study, in order to make these questions not too difficult or too easy for the students.

Factual knowledge questions required the recognition of specific information mentioned in the texts. For instance, one of the detail questions was: “In what year did U-boat operations reach American coastal waters? A. 1940, B. 1942, C. 1944, D. never”. Inference/comprehension questions required understanding and manipulating the acquired information from the texts. For example, such a question was: “You are the first Norse person to arrive in Finland. What was most likely your profession? A. Chieftain, B. Norwegian Royalty, C. Unsuccessful farmer, D. Viking naval officer”. The open-ended questions: “Describe, using one example from the text, how German forces successfully implemented naval warfare tactics to their advantage in the early 1940s”. The open questions were scored on basis of a standard correct answer.

Participants received 1 point per correct multiple-choice question and 2 points per correct open-ended question. This added up to a total of 72 points a participant could get for the exam. For the analysis the scores were also subdivided per question category: factual multiple-choice question (max 24 points), inference/comprehension multiple-choice questions (max 24 points), and open-ended questions (max 24 points). In addition, a final grade was calculated based on the total score to examine whether the number of participants that passed the test differed between the treatment groups. We used the grading system that is in place at Maastricht University, the Netherlands: a grade of 5.5 out of 10 is considered as a pass.

### Digit span task

Because some studies showed that baseline working memory performance can modulate the effects of MPH (Mattay et al. 2000; Mehta et al. 2000; van der Schaaf et al. 2013), a training session included the digit span task (forward and backward). Unfortunately, data were lost due to issues with data storage. The final dataset consisted of 60 participants (PLA-PLA, *n* = 22; MPH -PLA, *n* = 18; PLA-MPH, *n* = 20). The score of the forward and backward digit span were averaged per participant.

### Statistical analysis

Before the data were analyzed (using SPSS version 28.0.1.0), the data were checked for outliers and normality. All data were normally distributed. The blood pressure (systolic and diastolic) and heart rate data were analyzed with a GLM repeated measures ANOVA using Testing (before and after treatment) and Day as a within-subjects factor and Group as a between-subjects factor. One outlier was identified and removed from the data set (more than 2 standard deviations different). The effects of treatment on exam performance was tested per question category (multiple-choice factual knowledge, multiple-choice inference, open-ended question inference) using a one-way ANOVA in which the three groups were compared. This analysis was also applied for testing the total score and the grades. Partial eta squared effect sizes were calculated for the cardiovascular and exam questions analyses. The ANOVA analysis for the exam performance measures was complemented with a Bayesian analysis, testing whether the H0 and H1 hypothesis were considered similar or different based on the Bayes factor (JZS method). Differences in the number of students passing the exam was analyzed using a Fischer exact test, where the PLAC-PLAC group was compared with the other two groups separately. Cramer’s V effect size was calculated for the pass-fail analyses. Finally, a multivariate analysis was done using the digit span performance as a covariate in the model.

## Results

**Fig. 1:**
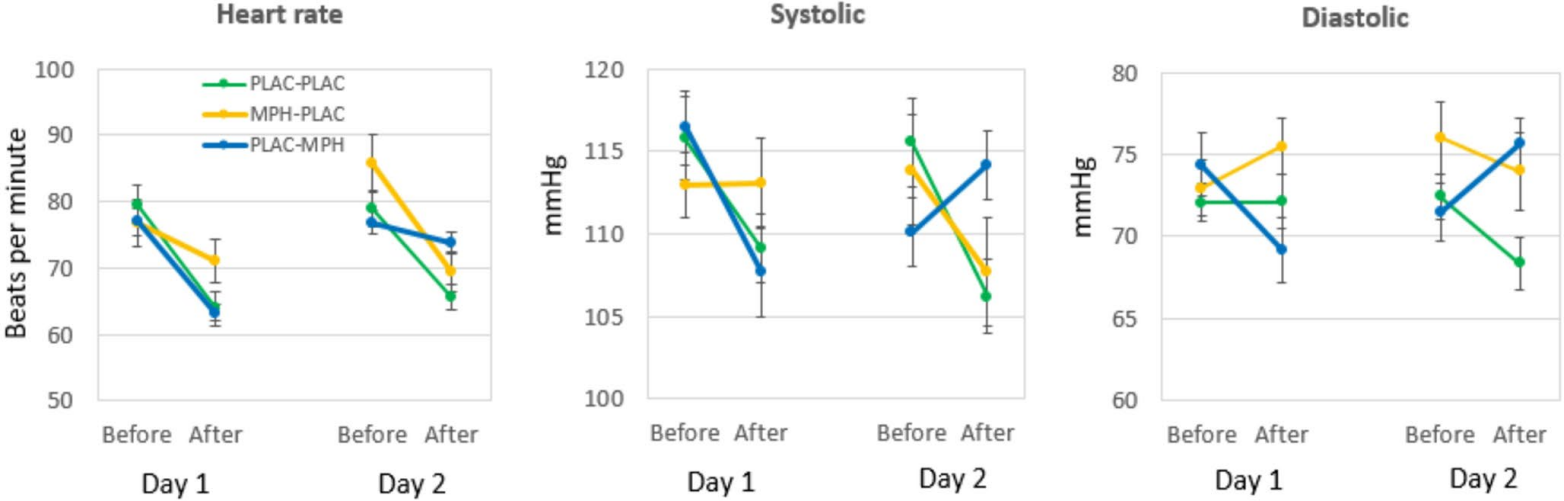
Effects of MPH on heart rate, systolic- and diastolic blood pressure before and after MPH treatment on day 1 (learning the text) and day 2 (exam). Data represent means and SEM

There was a three-way interaction (Group*Day*Treat) for heart rate, indicating that MPH had a different effect in the treatment groups on the two days (F(2,68) = 8.51, *p* < 0.001, *η*^2^ = 0.20). As can be seen in Fig. 1, this was mainly caused by a lower reduction in heart rate on day 1 for the MPH -PLA group and on day 2 for the PLA-MPH group. A similar three-way interaction effect was found for the systolic- and diastolic blood pressure data. On day 1, the systolic blood pressure did not decrease in the MPH -PLAC group and on day 2 the blood pressure only increased in the PLAC-MPH group (F(2,69) = 9.92, *p* < 0.001, *η*^2^ = 0.23). The diastolic blood pressure increased only in the MPH -PLAC group on day 1 and increased only in the PLAC-MPH group on day 2 (F(2,70) = 6.43, *p* < 0.003, *η*^2^ = 0.15).

### Exam performance

**Fig. 2:**
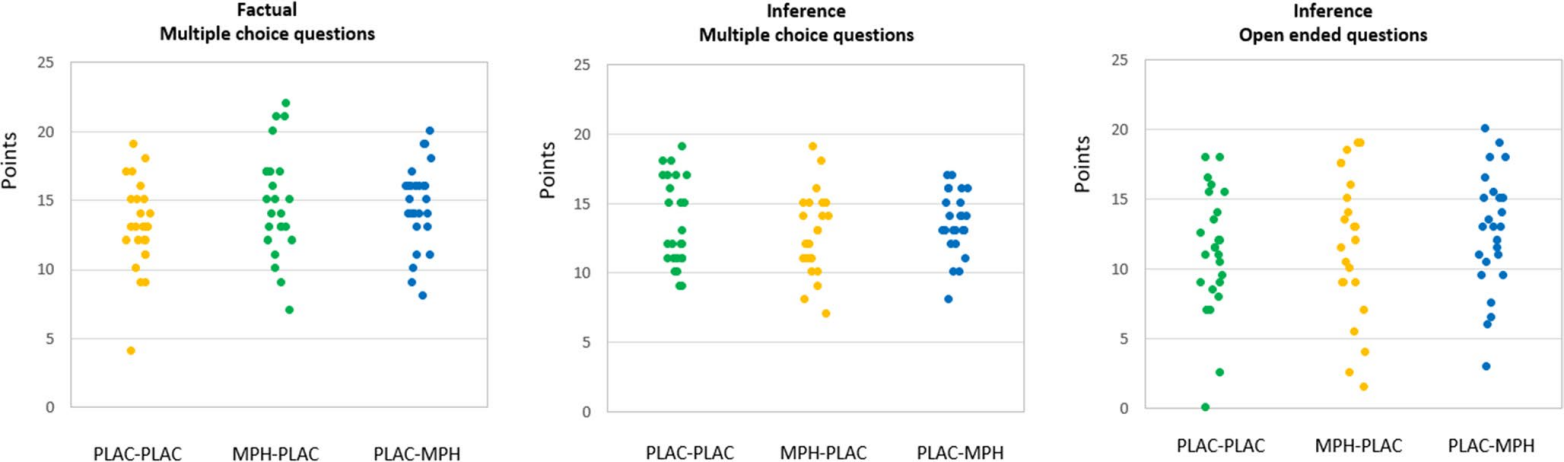
Performance (as shown by individual data points of participants) of the different groups on different types of exam questions

The descriptive data are shown in Table 1. As can be seen in Fig. 2, there was no difference between the treatment groups on the performance of factual multiple choice questions (F(2,72) = 1.89, *p* > 0.10, *η*^2^ = 0.05), inference multiple choice questions (F(2,72) = 0.67, *p* > 0.10, *η*^2^ = 0.02), and inference open questions (F(2,72) = 0.73, *p* > 0.10, *η*^2^ = 0.02). The Bayes factors ranged between 0.025 and 0.080, indicating that there was no evidence to reject the H0.

**Table 1:** Exam scores of the type of exam questions and grades. Data represent means (SEM)

	PLAC-PLAC	MPH-PLAC	PLAC-MPH
MC Factual	13.1(0.64)	14.8(0.78)	14.6(0.59)
MC Inference	13.5(0.63)	12.7(0.60)	13.5(0.43)
OE Inference	11.2(0.88)	11.94(1.08)	12.8(0.82)
Total points	37.8(1.75)	39.4(2.20)	40.9(1.63)
Grade	6.4(0.29)	6.6(0.35)	7.0(0.29)

**Fig. 3:**
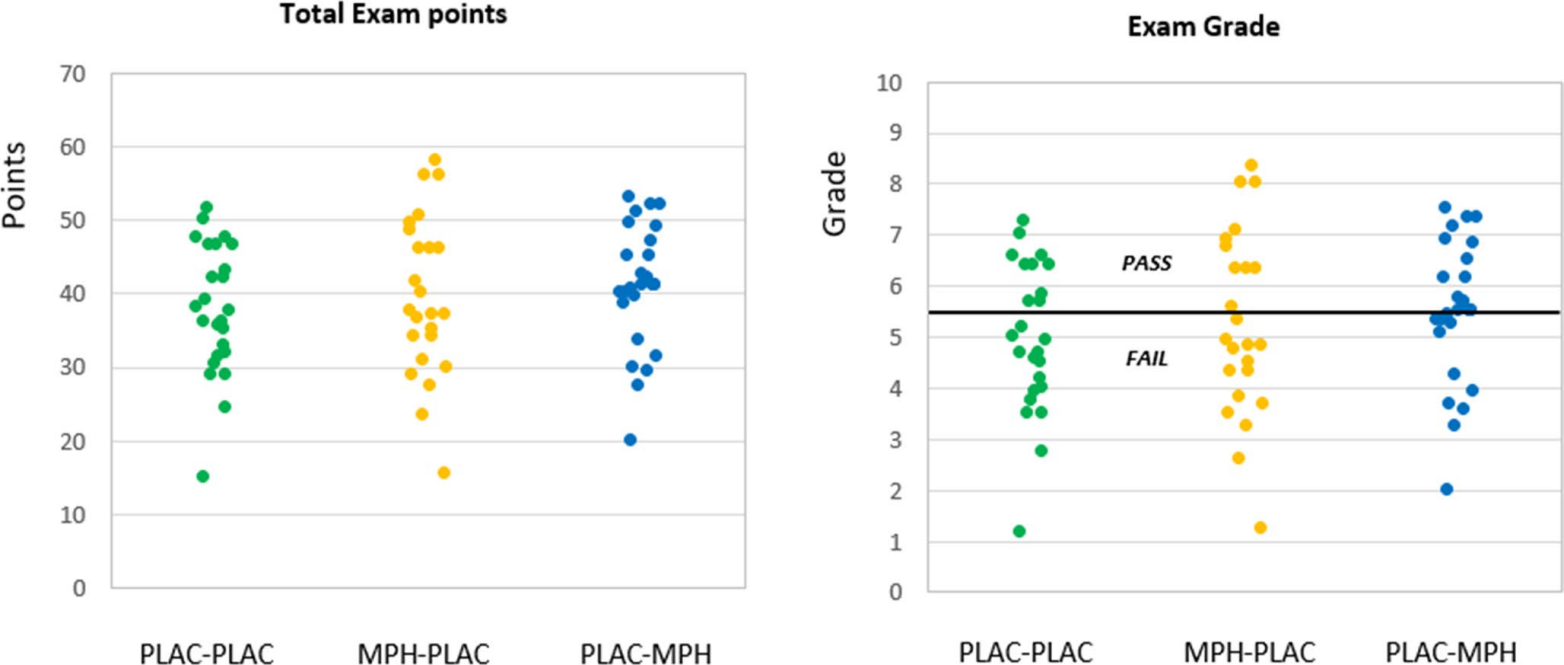
Individual data of total exam points and final exam grade of the students in the three treatment groups

When considering the total exam points and final grades, no group differences were found (see Fig. 3; F(2,72) = 0.70, *p* > 0.10, *η*^2^ = 0.02 and F(2,72) = 0.96, *p* > 0.10, *η*^2^ = 0.03, respectively). The Bayes factor was 0.026, indicating that there was no evidence to reject the H0. The number of students passing the exam was not different when the PLAC-PLAC group (10 pass, 15 fail) was compared with the MPH -PLAC group (10 pass, 14 fail; *X*^2^ = 0.42, *p* > 0.10, Cramer’s V = 0.03). Also, when comparing the PLAC-PLAC with the PLAC-MPH group (15 pass, 12 fail), no difference between the groups was found (*X*^2^ = 0.27, *p* > 0.10, Cramer’s V = 0.10).

### Digit span task

The multivariate analysis using the digit span performance as a covariate did not show a different outcome (Group: all F(2,59) < 1.86, *p* > 0.10, *η*^2^ < 0.04), indicating that the digit span performance did not influence the performance on the sub scores and total scores of the exam.

## Discussion

The aim of this study was to examine whether MPH could improve exam performance of students by giving MPH before studying or before the exam. Based on the data, MPH did not improve the exam performance. There was no indication that MPH specifically improved factual knowledge questions or inference questions. In addition, the number of students passing the exam was not different between the students who received MPH and the PLAC-PLAC group. The effects of MPH on the cardiovascular measures indicated that MPH had the expected effect on blood pressure and heart rate in on day 1 in the MPH-PLAC group and on day 2 in the PLAC-MPH group (e.g., Kimko et al. 1999).

This is the first study that specifically examined the effects of MPH on exam performance in healthy students. Previous studies in which the effects of MPH on cognition were examined measured the performance in tasks assessing specific aspects of cognitive performance in neuropsychological tests. Although MPH has shown to consistently improve working memory and processing speed (Linssen et al. 2014), these effects do not seem to translate into a better exam performance. It could be argued that better working memory may be helpful when learning factual knowledge, but this was not found in the current study. A relation between working memory and school performance has been reported in young children (e.g., Swanson and Alloway 2012), but this relation is very weak for high school or university students (e.g. Dubuc et al. 2020; Ishak et al. 2012). There appears to be a relation between working memory capacity and specific knowledge domains (e.g. mathematics and chemistry, see Opdenacker et al. 1990; Wang and Kao 2022). In the current study, the effects of MPH on exam performance were tested using more general knowledge texts. Whether MPH could improve the performance in domains that are more dependent on working memory (e.g., mathematics or chemistry), needs to be investigated in further studies.

The main question in this study was whether MPH could improve encoding/consolidation or retrieval of newly acquired information. Therefore, we did not include a group in which MPH was given before learning and before writing the exam. Based on the available literature, MPH seems to mainly affect consolidation (Linssen et al. 2014; Wagner et al. 2017). However, it should be mentioned that there are no studies that specifically investigated the effects of MPH retrieval in humans (i.e., administering MPH before the retrieval phase). Some animal studies tested the effects of MPH in fear condition and found that when MPH was injected before the extinction trial, memory extinction was impaired (e.g. Arellano Perez et al. 2020). This suggests that MPH may also affect retrieval processes. Although the effects of MPH on retrieval have not been investigated yet, the current study indicates that taking MPH before the exam does not improve exam performance.

The effects of MPH on academic performance have been investigated in children with attention deficit disorders. One early double-blind placebo controlled study showed that MPH improved cognitive (i.e., laboratory tasks) and academic (e.g., attention, self-correction) performance in children with attention deficit disorder with hyperactivity (Douglas et al. 1986; Murray et al. 2011). A meta-analysis showed that the academic achievements of children with attention deficit disorders were only modestly improved as compared to the improvements in symptoms (de Faria et al. 2022; Kortekaas-Rijlaarsdam et al. 2019). Clearly, these effects were evaluated after chronic treatment in children with attention deficit disorders, which is different from the acute administration in healthy participants in the current study. However, chronic MPH does improve cognitive performance in different domains in children with attention deficit disorders (Isfandnia et al. 2024), also lending support for the notion that improving cognitive functions does not necessarily translate to improved academic performance. In addition, it should be noted that the effects of acute MPH administration is different for attention deficit disorders patients and healthy volunteers (Coghill et al. 2014; Pievsky and McGrath 2018; Vertessen et al. 2022).

It has been suggested that the effects of MPH are dependent on level of endogenous catecholamine levels (Cools and D’Esposito 2011). This has been suggested based on imaging studies and working memory performance after MPH treatment. For example, it was found that the effects of MPH treatment on working memory was the strongest in those participants with lower baseline working memory performance (Mehta et al. 2000). In another study, it was shown that MPH did not improve associative memory but that there was an association with baseline working memory performance and activation in the right postcentral gyrus (Wagner et al. 2017). The notion that baseline working memory performance can predict the effects of MPH in individual participants was also found in a reward-punishment learning task, where poor working memory at baseline predicted a better performance in the reward-punishment learning task (van der Schaaf et al. 2013).

In contrast to these findings, we did not observe a relation between the different measures of exam performance and baseline digit span performance. Although it could be argued that this contrasts previous findings, it could also be argued that the exam performance is not dependent on central mechanisms affected by MPH. Thus, the relation between baseline working memory and task performance in previous studies was found in tasks that also seem to be sensitive to MPH treatment. This further supports the idea that exam performance appears to be independent of the central mechanisms affected by MPH, and that exam performance cannot be improved with MPH.

In this study, we used different texts of which the content was assumed not to be familiar with the participants of this study, i.e., Psychology students. Although we assumed that these topics were unfamiliar, it cannot be excluded that some students had some knowledge of the topics in these exam texts. We did not ask the students whether they were familiar with these topics. Another factor that may have led to individual differences is the motivation to learn the texts and perform on the exam. Although all participants were explicitly asked to perform as good as possible, the true level of motivation cannot be determined in this type of studies. Clearly, there is more at stake when they have to take an official exam. It is assumed that the randomization procedure controlled for the pre-existing knowledge of the texts and the level of motivation. Finally, it should also be noted that in this study, the time to study and the exam setting (individual in this study vs. group in official exams) are different from an official exam.

In the current study, only University students were selected to participate in this study. It could be argued that this population could already perform very well, and that methylphenidate could not further improve their performance. However, the duration that the students could study the texts and the complexity of the texts were piloted before the study was conducted, in order to prevent a very good or a very poor performance (i.e., ceiling or floor effect, respectively). When examining the data in the Figs. 2 and 3 it can be seen that there was sufficient room for improving the exam performance. It should also be noted that in earlier studies, in which also University students were included studies that examined the effect of MPH on verbal word learning, did show improvements in performance (see Linssen et al. 2014). Therefore, it is not very likely that the lack of effect of MPH in the current study was due to a ceiling effect.

In conclusion, this is a first study that aimed to test the effects of MPH on exam performance in healthy participants. This study showed that MPH did not improve exam performance when given before learning or before writing an exam. This should discourage students to take MPH during examinations since there are no benefits and only potential adverse effects. Moreover, this would annul the possible ethic issues surrounding the use of CEDs since academic performance seems not to be improved by taking MPH. However, further studies could investigate whether MPH could enhance performance in other knowledge domains, such as chemistry or mathematics, because these domains seem to relate to working memory capacity. In addition, the effects of other drugs, such as modafinil, on exam performance should be investigated. These type of studies are required in order to take a clear stand with respect to weighing the potential ethical issues and potential harmful effects of these drugs.

## Data Availability

Data are available upon reasonable request from the corresponding author.
